# International Comparisons of Fetal and Neonatal Mortality Rates in High-Income Countries: Should Exclusion Thresholds Be Based on Birth Weight or Gestational Age?

**DOI:** 10.1371/journal.pone.0064869

**Published:** 2013-05-20

**Authors:** Ashna D. Mohangoo, Béatrice Blondel, Mika Gissler, Petr Velebil, Alison Macfarlane, Jennifer Zeitlin, the Euro-Peristat Scientific Committee

**Affiliations:** 1 Department of Child Health, TNO, Netherlands Organization for Applied Scientific Research, Leiden, The Netherlands; 2 Epidemiological Research Unit on Perinatal and Women's and Children's Health, INSERM UMRS 953, Université Pierre-et-Marie Curie Paris6, Paris, France; 3 Information Department, National Institute for Health and Welfare, Helsinki, Finland; 4 Nordic School of Public Health, Gothenburg, Sweden; 5 Institute for the Care of Mother and Child; Perinatal Centre, Prague, Czech Republic; 6 Maternal and Child Health Research Centre; City University London, London, United Kingdom; 7 UPMC University Paris 06, Paris, France; National Institute of Child Health and Human Development, United States of America

## Abstract

**Background:**

Fetal and neonatal mortality rates are essential indicators of population health, but variations in recording of births and deaths at the limits of viability compromises international comparisons. The World Health Organization recommends comparing rates after exclusion of births with a birth weight less than 1000 grams, but many analyses of perinatal outcomes are based on gestational age. We compared the effects of using a 1000-gram birth weight or a 28-week gestational age threshold on reported rates of fetal and neonatal mortality in Europe.

**Methods:**

Aggregated data from 2004 on births and deaths tabulated by birth weight and gestational age from 29 European countries/regions participating in the Euro-Peristat project were used to compute fetal and neonatal mortality rates using cut-offs of 1000-grams and 28-weeks (2.8 million total births). We measured differences in rates between and within countries using the Wilcoxon signed rank test and 95% confidence intervals, respectively.

**Principal Findings:**

For fetal mortality, rates based on gestational age were significantly higher than those based on birth weight (*p*<0.001), although these differences varied between countries. The use of a 1000-gram threshold included 8823 fetal deaths compared with 9535 using a 28-week threshold (difference of 712). In contrast, the choice of a cut-off made little difference for comparisons of neonatal deaths (difference of 16). Neonatal mortality rates differed minimally, by under 0.1 per 1000 in most countries (*p* = 0.370). Country rankings were comparable with both thresholds.

**Conclusions:**

Neonatal mortality rates were not affected by the choice of a threshold. However, the use of a 1000-gram threshold underestimated the health burden of fetal deaths. This may in part reflect the exclusion of growth restricted fetuses. In high-income countries with a good measure of gestational age, using a 28-week threshold may provide additional valuable information about fetal deaths occurring in the third trimester.

## Introduction

There is an ongoing debate about the value of international comparisons of fetal and neonatal mortality rates, given differences between countries in recording of births and deaths at borderline viability [1], [2]. Fetal and neonatal mortality rates are highly sensitive to these inclusion criteria [1], [3], [4]. Differences in recording criteria are most acute for fetal deaths [5]. These deaths are recorded from as early as 16 completed weeks of gestation in Norway or 20 completed weeks in the United States to 26 completed weeks in Italy and Spain [3], [5]. Denmark and Sweden recorded fetal deaths beginning at 28 completed weeks until 2004 and 2008, respectively. While only a small proportion of births occur before 24 completed weeks of gestation (about 1 per 1000) [6], survival is rare and most of them are either fetal deaths or live births followed by a neonatal death. These births have a substantial impact on perinatal mortality statistics [3]. Valid analyses of fetal and neonatal mortality across countries thus require specifying common inclusion limits. These criteria are based either on gestational age, or on birth weight or on a combination of these.

The World Health Organization (WHO) recommends the use of a 1000-gram threshold for international comparisons of perinatal mortality rates [7]. This limit makes it possible to provide a measure of the health burden of third trimester perinatal deaths, since 1000 grams corresponds approximately to the birth weight at 28 completed weeks of gestation, the beginning of the third trimester. This measure provides only a partial view of overall mortality, since a large proportion of deaths in high-income countries (between 25–60%) occur to babies born in the second trimester [3], [6], but using this threshold has the benefit of enabling greater comparability between countries. Participants in a recent international collaboration on stillbirths agreed that an analysis of third trimester deaths has public health relevance for international comparisons in high-income countries [8].

The aim in international comparisons is to maximise both comparability and scientific and policy relevance. The primary aim of using a birth weight threshold for international comparisons is to ensure comparability because birth weight measures are considered to be less prone to error than calculations of gestational age. When the date of the last menstrual period (LMP) is used alone to calculate gestational age, the results can be inaccurate [9], especially if the woman has no antenatal care or if antenatal care starts late in pregnancy. However, most high-income countries use a clinical estimate of gestational age that incorporates information from dating ultrasounds and is therefore of better quality [10]. Birth weight data also have limitations since babies who are stillborn or die before they can be transferred to a neonatal unit may not be systematically weighed [11].

Gestational age is generally considered to be a more relevant variable than birth weight for studying perinatal outcomes. Recent European cohorts have analysed the outcome for very preterm rather than very low birth weight babies, as gestational age has a better prognostic value [12]–[16]. Furthermore, when obstetricians are making decisions during pregnancy, they have reasonably precise information about gestational age but not about birth weight. Finally, birth weight distributions differ between and within populations and European comparisons have found that the birth weight at which mortality is lowest varies between European countries [17]. Using birth weight cut-offs will exclude relatively more births and deaths in countries where average birth weights are lower and this may introduce bias.

While the hypothesis underlying the current WHO recommendation is that the 1000-gram threshold provides a good approximation for the 28^th^ week of gestation or the beginning of the third trimester, this hypothesis has not been tested. The aim of this analysis was therefore to compare the use of a 1000-gram birth weight threshold with a 28-week gestational age threshold in terms of their impact on reporting of fetal and neonatal mortality rates within European countries and on comparisons between European countries.

## Methods

This study was embedded within Euro-Peristat, which developed a list of valid and reliable indicators for monitoring and evaluating perinatal health in the European Union (EU) [18]. Twenty-five EU member states and Norway participated. Detailed information on the design and methods is available elsewhere [5], [19], [20]. National population-based data for each indicator for the year 2004 were requested in aggregated form from members of the Euro-Peristat Scientific Committee. If national data were not available, population-based regional data could be provided instead.

The Euro-Peristat core indicator list includes fetal and neonatal mortality. The fetal mortality rate is defined as the number of deaths before or during birth in a given year per 1000 live and stillbirths in the same year. The neonatal mortality rate is defined as the number of deaths at 0 to 27 days after live birth in a given year per 1000 live births in the same year. Euro-Peristat collects data on births and deaths at or after 22 weeks of gestation, regardless of birth weight. Aggregated data on the number of live births, fetal and neonatal deaths by each week of gestation and by birth weight intervals of 500 grams were collected. These data were used to calculate fetal and neonatal mortality rates for births and deaths weighing 1000 grams and over and for those born at or after 28 completed weeks.

Twenty-seven countries were able to provide data to calculate fetal mortality rates with birth weight and gestational age thresholds. Germany, Hungary, Ireland and Italy did not have data on neonatal deaths by birth weight or gestational age. Some countries could only provide data on some regions (Valencia in Spain, Brussels and Flanders in Belgium). Data from France came from a one-week national perinatal survey in October 2003, vital registration, and neonatal death certificates. Data on neonatal deaths from England and Wales related to 2005 and data from Italy were for 2003. Table S1 presents additional information about the data sources. These constraints reflect the diversity of sources for perinatal health data in Europe [5].

### Missing Data

Most countries had fewer than 5% of data missing for fetal and neonatal deaths by birth weight and gestational age, as presented in Table S2. However, there were some exceptions. The percentages of fetal deaths with birth weights missing were 30.7% in Denmark, 25.0% in Italy, 22.7% in Brussels, 13.9% in Valencia, 6.4% in Portugal, 5.9% in Luxembourg, and 5.1% in France. Gestational age was missing for 17.0% of fetal deaths in Brussels, 11.7% in Valencia and 9.5% in Portugal. We excluded countries where the proportion of fetal deaths with missing birth weight was significantly different from the proportion with missing gestational age. These were Denmark (30.7% of birth weights vs. 4.2% of gestational ages) and Italy (25.0% of birth weights and 0% for gestational age). These divergent proportions of missing data would have biased our ability to compare rates.

For neonatal deaths, fewer countries had high proportions of data missing. Over 5% of birth weights were missing for Denmark (14.8%), Luxembourg (9.1%), Sweden (7.1%), Scotland (6.8%), and Valencia (5.8%). Gestational ages were missing for over 5% in Luxembourg (9.1%), Denmark (7.0%), Portugal (6.8%), and Valencia (6.8%). As with fetal deaths, we excluded countries with highly divergent proportions of missing birth weights and gestational ages. We therefore excluded Denmark (14.8% of birth weights vs. 7.0% of gestational ages) and Sweden (7.1% of birth weights and 0% for gestational age). Live birth data were missing for less than 5% with the exception of Brussels and Valencia where 6.3% and 5.5% of gestational ages were missing respectively.

For countries included in the analyses, we excluded missing data from our primary analyses as this would reflect the reality if these cut-offs were used, but we also did a second set of analyses with missing data distributed according to observed birth weight and gestational age distributions for live births, and fetal and neonatal deaths separately.

### Statistical Analysis

We calculated fetal and neonatal mortality rates with 95% confidential intervals, using both birth weight and gestational age thresholds. We also computed differences between rates with confidence intervals to test whether these were significant within countries. To test whether there was a systematic difference between countries in rates based on birth weight versus gestational age we used the non-parametric Wilcoxon signed rank test. In addition, we used the Wilcoxon rank sum test to assess whether rates based on a 28-week threshold minus a 1000-gram threshold differed significantly across countries. Finally, we tested the correlation between these two rates using the Spearman rank test to assess how these affected country rankings. These statistical tests were repeated on recalculated rates after imputation of missing observations, as described above, to ensure that the addition of these data would not change our results. Analyses were done with SPSS version 17.0 for Windows (SPSS Inc, Chicago, IL, USA).

## Results

Table 1 presents fetal and neonatal mortality rates using a birth weight cut-off of 1000 grams. The range for fetal deaths was 1.6 to 4.7 per 1000 live and stillbirths and the range of neonatal deaths was 1.1 to 4.3 per 1000 live births. Also shown are the same rates with a gestational age cut-off of 28 weeks. They ranged from 1.7 to 4.9 per 1000 for fetal deaths and 1.3 to 4.0 per 1000 for neonatal deaths.

**Table 1:** Fetal mortality rates per 1000 total births and neonatal mortality rates per 1000 live births with 95% confidence intervals [CI].

	Fetal mortality				Neonatal mortality			
	Birth weight ≥1000 grams		Gestational age ≥28 weeks		Birth weight ≥1000 grams		Gestational age ≥28 weeks	
Country/region	Total births	Rate [95% CI]	Total births	Rate [95% CI]	Live births	Rate [95% CI]	Live births	Rate [95% CI]
Austria	78820	2.33 [2.0–2.7]	78794	2.49 [2.1–2.8]	78636	1.44 [1.2–1.7]	78598	1.39 [1.1–1.6]
Belgium: Brussels	15752	2.54 [1.8–3.3]	15176	3.36 [2.4–4.3]	15712	1.91 [1.2–2.6]	15125	2.18 [1.4–2.9]
Belgium: Flanders	60642	2.67 [2.3–3.1]	60679	2.85 [2.4–3.3]	60480	1.37 [1.1–1.7]	60506	1.39 [1.1–1.7]
Czech Republic	97544	2.56 [2.2–2.9]	97480	2.40 [2.1–2.7]	97294	1.12 [0.9–1.3]	97365	1.25 [1.0–1.5]
Estonia	13945	3.37 [3.3–4.3]	13939	3.16 [2.2–4.1]	13898	2.52 [1.7–3.4]	13895	2.66 [1.8–3.5]
Finland	57482	1.97 [1.6–2.3]	57407	2.04 [1.7–2.4]	57369	1.20 [0.9–1.5]	57290	1.29 [1.0–1.6]
France	14551	4.12 [3.1–5.2]	14540	4.88 [3.8–6.0]	761290	1.50 [1.4–1.6]	765752	1.48 [1.4–1.6]
Germany	644654	2.39 [2.3–2.5]	645401	2.55 [2.4–2.7]				
Hungary	94801	3.55 [3.2–3.9]	94900	3.73 [3.3–4.1]				
Ireland	62077	3.82 [3.3–4.3]	62097	4.28 [3.8–4.8]				
Latvia	20393	4.71 [3.8–5.6]	20382	4.86 [3.9–5.8]	20297	4.34 [3.4–5.2]	20283	3.99 [3.1–4.9]
Lithuania	29510	3.83 [3.1–4.5]	29502	3.93 [3.2–4.6]	29397	2.89 [2.3–3.5]	29386	2.93 [2.3–3.5]
Luxembourg	5296	2.45 [1.1–3.8]	5384	2.79 [1.4–4.2]	5283	1.51 [0.5–2.6]	5369	1.30 [1.7–5.5]
Malta	3889	3.86 [1.9–5.8]	3894	3.85 [1.9–5.8]	3874	3.36 [1.5–5.2]	3879	3.61 [1.7–5.5]
The Netherlands	181014	3.77 [3.5–4.1]	178710	4.27 [4.0–4.6]	180332	1.96 [1.8–2.2]	177947	1.93 [1.7–2.1]
Norway	57450	2.75 [2.3–3.2]	57004	2.84 [2.4–3.3]	56911	1.32 [1.0–1.6]	56925	1.35 [1.1–1.7]
Poland	356571	3.54 [3.3–3.7]	356734	3.77 [3.6–4.0]	355307	2.92 [2.7–3.1]	355389	3.00 [2.8–3.2]
Portugal	108948	2.64 [2.3–2.9]	109136	2.69 [2.4–3.0]	108660	1.51 [1.3–1.7	108842	1.45 [1.2–1.7]
Slovenia	17840	3.48 [2.6–4.3]	17849	3.53 [2.7–4.4]	17778	1.29 [0.8–1.8]	17786	1.35 [0.8–1.9]
Slovak Republic	52301	1.63 [1.3–2.0]	52332	1.66 [1.3–2.0]	52216	1.63 [1.3–2.0]	52245	1.70 [1.3–2.1]
Spain: Valencia	49505	2.95 [2.5–3.4]	48279	3.11 [2.6–3.6]	49359	1.22 [0.9–1.5]	48129	1.25 [0.9–1.6]
Sweden	99928	2.87 [2.5–3.2]	100111	3.16 [2.8–3.5]				
UK: England and Wales	637653	3.68 [3.5–3.8]	637521	4.13 [4.0–4.3]	640374	1.59 [1.5–1.7]	637521	1.59 [1.5–1.7]
UK: Northern Ireland	22351	3.62 [2.8–4.4]	22355	3.76 [3.0–4.6]	22270	1.53 [1.0–2.0]	22271	1.44 [0.9–1.9]
UK: Scotland	52907	4.06 [3.5–4.6]	52860	4.58 [4.0–5.2]	52692	1.54 [1.2–1.9]	52618	1.48 [1.2–1.8]

Cyprus and Greece (no data on fetal and neonatal death by birth weight and gestational age), Germany, Hungary, Ireland and Italy (no data on neonatal death by birth weight and gestational age), Denmark and Italy (excluded from fetal death comparisons, because of highly divergent missing data on birth weight versus gestational age), Denmark and Sweden (excluded from neonatal death comparisons, because of highly divergent missing data on birth weight versus gestational age).

**Figure 1:**
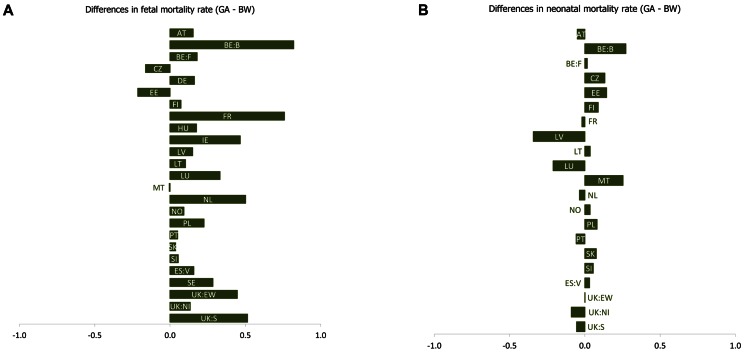
Differences in mortality rates based on gestational age ≥28 weeks minus birth weight ≥500 grams. Austria (AT), Brussels (BE: BR), Flanders (BE: FL), Cyprus (CY), Czech Republic (CZ), Denmark (DK), Estonia (EE), Finland (FI), France (FR), Germany (DE), Greece (GR), Hungary (HU), Ireland (IE), Italy (IT), Latvia (LV), Lithuania (LT), Luxembourg (LU), Malta (MT), the Netherlands (NL), Norway (NO), Poland (PL), Portugal (PT), Slovenia (SI), Slovak Republic (SK), Valencia region of Spain (ES), Sweden (SE), and the United Kingdom (UK): England and Wales combined (UK: EW), Northern Ireland (UK: NI), and Scotland (UK: SC).

Except for the Czech Republic (0.16‰) and Estonia (0.21‰), where rates were 0.2 per 1000 higher with a birth weight cut-off, most countries had higher rates of fetal deaths when a gestational age cut-off was used, as illustrated in Figure 1. For seven out of 25 countries/regions, the two rates were very similar with minimal differences of 0.1 per 1000 or less. The widest differences were 0.8 per 1000 in Brussels (0.82‰) and France (0.76‰). At an individual country level, differences between fetal mortality rates based on gestational age and those based on birth weight were not significantly different from zero, except in the Netherlands where the difference was 0.50 per 1000 with 95% confidence interval 0.09–0.91 (*p* = 0.018) and England and Wales where the rate difference was 0.45 per 1000 with 95% confidence interval 0.23–0.66 (*p*<0.001).

In contrast, differences between neonatal mortality rates were minimal, with 15 out of 21 countries/regions having differences between −0.1 and +0.1 per 1000 (Figure 1). Rates calculated with a gestational age cut-off were not significantly higher or lower than those with a birth weight cut-off, although in Latvia (−0.35‰), Brussels (+0.27‰) and Malta (+0.25‰) differences were 0.25 per 1000 or more.

Differences between countries in fetal mortality rates based on gestational age compared with those based on birth weight were significant (*p*<0.001 for Wilcoxon signed rank test). The corresponding neonatal mortality rates did not differ significantly between countries (*p* = 0.370), however twelve countries had a positive difference while eight had a negative and there was one tie. In total, 8823 fetal deaths were included when a 1000-gram threshold was used compared with 9535 with a 28-week threshold, a difference of 712 fetal deaths (7.5% of all fetal deaths). In contrast, the difference in neonatal deaths was minimal, 4710 using a 1000-gram threshold versus 4726 using a 28-week threshold (a difference of 16).

Results did not change when the observed birth weight and gestational age distributions for fetal and neonatal deaths and live births were used to include births and deaths with missing birth weights and gestational ages in the analyses. Fetal mortality rates based on gestational age were still significantly higher than those based on birth weight (*p* = 0.002); while the choice of a cut-off made no difference for comparisons of neonatal mortality rates (*p* = 0.380).

Fetal and neonatal mortality rates computed using a birth weight threshold were highly correlated with rates computed using a gestational age threshold, with Spearman rank correlations of ρ = 0.952 (*p*<0.001, n = 25) for fetal mortality and ρ = 0.963 (*p*<0.001, n = 21) for neonatal mortality. Country rankings were therefore similar with a few exceptions such as Brussels which ranked sixth for birth weight and thirteenth for gestational age. Even for the Netherlands and England and Wales, where differences in fetal mortality rates calculated using the two definitions were significantly different, their ranks only differed by two places (19 and 18 out of 25 for birth weight to 21 and 20 out of 25 for gestational age, respectively (data not shown in table).

## Discussion

Our analysis showed that fetal mortality rates in European countries were higher when based on a 28-week gestational age threshold compared with a 1000-gram birth weight threshold, whereas the choice of a threshold made little difference for neonatal mortality rates. These results suggest that a substantial proportion of fetal deaths occurring at or after 28 weeks of gestation have a birth weight under 1000-grams. Despite this difference, however, the selection of a cut-off did not change countries’ relative positions. The small differences between neonatal mortality rates calculated using birth weight and gestational age cut-offs and the similarity in rankings suggest that differences in average birth weight between populations do not create a bias when rates are computed using a birth weight cut-off.

There are a number of limitations to this study. Most notably its reliance on aggregated data meant we could not cross tabulate the birth weight and gestational age distributions of the excluded births and deaths. Our data also date from 2004 and practices in registration and care of very preterm infants may have changed since this time. However, these changes most likely occurred for births with a birth weight under 1000 grams or before 28 completed weeks of gestation which are excluded from our analysis. At the time these data were compiled, a widespread consensus in Europe existed about the importance of active care for infants born at or after 28 weeks of gestation or 1000 grams or more [21]. Furthermore, in all countries live and stillbirths born at these thresholds were included in routine data collection systems [5]–[7].

Another limitation relates to missing data; many countries had some birth weight and gestational age data missing and this will have affected absolute rates. While we excluded countries with highly divergent proportions of birth weight and gestational age data missing, some countries still had more data missing among deaths than among live births. Missing data could be more prevalent among extremely preterm or very low birth weight babies, which would limit their influence on analyses of rates using 28 weeks or 1000 grams thresholds. Because we were using aggregated data, we were limited in our ability to investigate this further. However, even if this were not the case, these missing data are unlikely to change our conclusions. This was shown when we repeated our analyses including missing data based on observed distributions of birth weight and gestational age for fetal and neonatal deaths and live births. Nonetheless, this analysis showed that proportions of missing gestational age and birth weight varied between countries and this may have an impact on the comparison of mortality rates when these thresholds are used, regardless of the choice of threshold. These proportions should be reported in comparative analyses.

Finally, while the Euro-Peristat project requests data based on the best obstetric estimate of gestational age in weeks from clinical records, it was not possible to evaluate differences in the ways in which participating countries actually measure gestational age. In most European countries, however, dating ultrasounds are a standard component of care during pregnancy and most women have their first antenatal visit in the first trimester [22]–[24].

Using a birth weight cut-off of 1000 grams resulted in 712 fewer fetal deaths overall compared with using a cut-off of 28 completed weeks of gestation leading to systematically lower fetal death rates. A previous review also suggested that stillbirth rates were higher using gestational age limits based on Norwegian data showing that a 500 grams cut-off point excluded more stillbirths than a 22 week cut-off point; neonatal deaths were not included in this study [4]. By comparing neonatal deaths with fetal deaths, our results show that this effect specifically relates to stillbirths and does not reflect the gestational age and birth weight distribution of all births.

There are several possible explanations for this finding. First, using a birth weight cut-off may exclude growth restricted fetuses. As concluded by a recent review of stillbirths in high-income countries, small for gestational age is the pregnancy condition with the highest population attributable risk (measured at one out of four for stillbirths) [25]. Fetal growth restriction is a particularly important risk factor for antepartum deaths, which constitute over 80% of fetal deaths in high-income countries [4]. Second, fetal weight loss after antepartum death may also contribute to lower birth weights, although the extent of this phenomenon is still unknown [26]. Finally, some deaths may predate delivery and this would lead to lower average birth weights for stillbirths. More detailed analysis of stillbirths with a gestational age of 28 weeks and over, but birth weights under 1000 grams is needed to better understand the relative contribution of these different explanations.

While growth restriction is also a risk factor for neonatal death, the magnitude of the association may be less strong, especially in the gestational age and birth weight bands considered in this analysis. Recent studies in France and New Zealand found that 17% and 13% of all neonatal deaths were below the tenth percentiles of national standards [27], [28]. This compared to studies of stillbirth where between 40% and 60% are associated with growth restriction [29]. Growth restriction in this context reflects a wide range of underlying pregnancy complications which contribute to poor growth and adverse perinatal outcomes.

### Conclusions

In the European countries included in our analysis, fetal mortality rates calculated using a threshold of 28 weeks of gestation were higher than those based on birth weight cut-offs of 1000 grams, probably due in part to the role of intra-uterine growth restriction in antepartum fetal deaths. Assessing the health burden of third trimester fetal deaths using a cut-off based on gestational age provides valuable additional information. Comparisons based on this cut-off are possible in countries where a clinical estimate of gestational age is recorded in routine data sources and where women have access to early antenatal care and dating ultrasound as is the case in European and other high-income countries.

## Supporting Information

Table S1 **Data sources used for data on live births, fetal and neonatal deaths in Europe in 2004.**
(DOCX)

Table S2 **Percentage of missing birth weights (BW) and gestational ages (GA).** *Denmark (fetal and neonatal mortality), Italy (fetal mortality) and Sweden (neonatal mortality) were excluded from analysis because of substantial difference in missing data by birth weight and gestational age. Proportions of 5% and over missing data are presented in bold.
(DOCX)
