# Supplementary material for: International Comparisons of Fetal and Neonatal Mortality Rates in High-Income Countries: Should Exclusion Thresholds Be Based on Birth Weight or Gestational Age?
Source: PLoS One. 2013 May 20;8(5):e64869. doi: 10.1371/journal.pone.0064869 (PMC3658983; doi:10.1371/journal.pone.0064869)
Supplement: Table S1 — Data sources used for data on live births, fetal and neonatal deaths in Europe in 2004. (DOCX) [file pone.0064869.s001.docx]

**Table S1. Data sources used for data on live births, fetal and neonatal deaths in Europe in 2004**

| **Country: Region** | **Data sources** |
| --- | --- |
| Austria | Statistics Austria (National Birth Statistics) |
| Belgium: Brussels | Linked birth and death certificates |
| Belgium: Flanders | Study Center for Perinatal Epidemiology (SPE) |
| Cyprus | Cyprus Statistical Service (CYSTAT), Vital Statistics |
| Czech Republic | Institute of Health Information and Statistics of Czech Republic (UZIS CR) |
| Denmark | Danish Perinatal Database |
| Estonia | Statistics Estonia, Estonian Medical Birth Registry, and Estonian Mortality Database |
| Finland | Medical Birth Register |
| France (2003) | National Perinatal Survey, Birth certificates, Neonatal Death Certificate |
| Germany | Medical Birth Register [www.bqs-online.de](http://www.bqs-online.de) |
| Greece | National database |
| Hungary | Hungarian Central Statistics Office |
| Ireland | National Perinatal Reporting System (NPRS) |
| Italy (2003) | National Birth Certificates Register |
| Latvia | Newborns Register of Latvia and Death Cause Data Base |
| Lithuania | Medical Data of Births |
| Luxembourg | FIMENA Fiche Médicale de Naissance and Mortality Statistics / Ministry of Health |
| Malta | National Mortality Register |
| The Netherlands | The Netherlands Perinatal Registry |
| Norway | Medical Birth Registry of Norway |
| Poland | Birth and death certificates |
| Portugal | Demographic Statistics - National Institute of Statistics |
| Slovak Republic | Report on newborn - Z (MZ SR) 5-12 |
| Slovenia | National Perinatal System of Slovenia |
| Spain: Valencia | Perinatal Mortality Register |
| Sweden | Medical Birth Register |
| United Kingdom: England and Wales (2005) | Civil registration of births and deaths/ NHS Numbers for Babies records |
| United Kingdom: Northern Ireland | The Confidential Enquiry into Maternal and Child Health (CEMACH)/ Child health systems |
| United Kingdom: Scotland | Scottish Stillbirth and Infant Death Enquiry |
