# Supplementary material for: International Comparisons of Fetal and Neonatal Mortality Rates in High-Income Countries: Should Exclusion Thresholds Be Based on Birth Weight or Gestational Age?
Source: PLoS One. 2013 May 20;8(5):e64869. doi: 10.1371/journal.pone.0064869 (PMC3658983; doi:10.1371/journal.pone.0064869)
Supplement: Table S2 — Percentage of missing birth weights (BW) and gestational ages (GA). *Denmark (fetal and neonatal mortality), Italy (fetal mortality) and Sweden (neonatal mortality) were excluded from analysis because of substantial difference in missing data by birth weight and gestational age. Proportions of 5% and over missing data are presented in bold. (DOCX) [file pone.0064869.s002.docx]

**Table S2 Percentage of missing birth weights (BW) and gestational ages (GA)**

|  | **Fetal deaths** | | **Neonatal deaths** | | **Live births** | |
| --- | --- | --- | --- | --- | --- | --- |
|  | **BW** | **GA** | **BW** | **GA** | **BW** | **GA** |
| Austria | 0.0 | 0.0 | 0.0 | 0.0 | 0.0 | 0.0 |
| Belgium: Brussels | **22.7** | **17.0** | 2.0 | 0.0 | 2.6 | **6.3** |
| Belgium: Flanders | 0.0 | 0.0 | 0.0 | 0.0 | 0.0 | 0.0 |
| Czech Republic | 0.0 | 0.0 | 0.0 | 0.0 | 0.0 | 0.0 |
| Denmark | **30.7** | 4.2 | **14.8** | **7.0** | 0.3 | 0.1 |
| Germany | 0.2 | 1.2 | NA | NA | NA | NA |
| Estonia | 0.0 | 0.0 | 0.0 | 1.7 | 0.3 | 0.3 |
| Finland | 0.0 | 2.6 | 4.3 | 1.4 | 0.0 | 0.2 |
| France (2003) | **5.1** | 0.6 | 15.2 | 12.0 | 0.5 | 0.4 |
| Hungary | 0.4 | 1.3 | NA | NA | NA | NA |
| Ireland | 0.6 | 0.0 | NA | NA | NA | NA |
| Italy (2003) | **25.0** | 0.0 | NA | NA | NA | NA |
| Latvia | 0.0 | 0.0 | 0.0 | 1.7 | 0.0 | 0.0 |
| Lithuania | 0.0 | 0.0 | 0.0 | 0.0 | 0.0 | 0.0 |
| Luxembourg | **5.9** | 0.0 | **9.1** | **9.1** | 3.4 | 1.8 |
| Malta | 0.0 | 0.0 | 0.0 | 0.0 | 0,1 | 0.0 |
| The Netherlands | 0.6 | 1.4 | 0.2 | 2.4 | 0.0 | 1.4 |
| Norway | 1.2 | 0.0 | 0.9 | 0.0 | 0.0 | 0.0 |
| Poland | 0.2 | 0.3 | 0.3 | 1.4 | 0.0 | 0.0 |
| Portugal | **6.4** | **9.5** | 2.9 | **6.8** | 0.3 | 0.2 |
| Slovak Republic | 0.0 | 0.0 | 0.0 | 0.0 | 0.0 | 0.0 |
| Slovenia | 0.0 | 0.0 | 0.0 | 0.0 | 0.0 | 0.0 |
| Spain: Valencia | **13.9** | **11.7** | **5.8** | **6.8** | 3.1 | **5.5** |
| Sweden | **3.8** | 0.0 | **7.1** | 0.0 | 0.2 | 0.1 |
| UK: England and Wales (2005) | 1.6 | 1.8 | 3.5 | 2.8 | 0.3 | 0.8 |
| UK: Northern Ireland | 0.0 | 0.0 | 0.0 | 0.0 | 0.0 | 0.0 |
| UK: Scotland | 1.1 | 0.0 | **6.8** | **5.0** | 0.0 | 0.2 |

Denmark (fetal and neonatal mortality), Italy (fetal mortality) and Sweden (neonatal mortality) were excluded

from analysis because of substantial difference in missing data by birth weight and gestational age.

Proportions of 5% and over missing data are presented in bold.
